# Pregnancy Associated Plasma Protein-A and Placental Growth Factor in a Sub-Saharan African Population: A Nested Cross-Sectional Study

**DOI:** 10.1371/journal.pone.0159592

**Published:** 2016-08-17

**Authors:** Joyce L. Browne, Kerstin Klipstein-Grobusch, Maria P. H. Koster, Dhivya Ramamoorthy, Edward Antwi, Idder Belmouden, Arie Franx, Diederick E. Grobbee, Peter C. J. I. Schielen

**Affiliations:** 1 Julius Global Health, Julius Center for Health Sciences and Primary Care, University Medical Center Utrecht, Utrecht, the Netherlands; 2 Division of Epidemiology & Biostatistics, School of Public Health, Faculty of Health Sciences, University of the Witwatersrand, Johannesburg, South Africa; 3 Department of Obstetrics and Gynecology, University Medical Center Utrecht, The Netherlands; 4 Ghana Health Service, Greater Accra Regional Health Directorate, Accra, Ghana; 5 Center for Infectious Diseases Research, Diagnostics and Screening (IDS), National Institute for Public Health and the Environment (RIVM), Bilthoven, The Netherlands; Stellenbosch University, SOUTH AFRICA

## Abstract

**Background:**

Baseline distributions of pregnancy disorders’ biomarkers PlGF and PAPP-A levels are primarily based on Western European populations of Caucasian ethnicity. Differences in PAPP-A and PlGF concentrations by ethnicity have been observed, with increased levels in Afro-Caribbean, East Asian, and South Asian women. Baseline concentrations of sub-Saharan African women have not been evaluated.

**Objectives:**

To investigate PlGF and PAPP-A in a sub-Saharan African population and assess the performance of existing reference values of PAPP-A and PlGF.

**Methods:**

A nested cross-sectional study was conducted in two public hospitals in Accra, Ghana. Out of the original 1010 women enrolled in the cohort, 398 participants were eligible for inclusion with a normotensive singleton gestation and serum samples taken between 56–97 days of pregnancy. PAPP-A and PlGF concentrations were measured with an automated immunoassay. Multiple of the median (MoM) values corrected for gestation and maternal weight for PAPP-A and PlGF were calculated using reference values of a Dutch perinatal screening laboratory based on over 10.000 samples, and PlGF manufacturer reference values, respectively.

**Results:**

The PAPP-A median MoM was 2.34 (interquartile range (IQR) 1.24–3.97). Median PlGF MoM was 1.25 (IQR 0.95–1.80). Median MoM values for PAPP-A and PlGF tended to be slightly different for various Ghanaian ethnic subgroups.

**Conclusions:**

PAPP-A and PlGF MoM values appear to be substantially higher in a sub-Saharan African population compared to the Caucasian or Afro-Caribbean MoM values previously reported. The difference suggests the need for a specific correction factor for this population to avoid underestimation of risk for fetal aneuploidies or placental disorders when using PAPP-A and PlGF MoM for screening purposes.

## Introduction

Reduced levels of pregnancy associated plasma protein A (PAPP-A) and placental growth factor (PlGF) around 8 to 14 weeks of gestational age are associated with obstetric complications such as preeclampsia, small-for-gestational age (SGA), intra-uterine growth restriction (IUGR) and stillbirth [1–3]. These markers perform best when combined with others in risk models including maternal characteristics and uterine artery pulsatility index (PI) [2,3]. PAPP-A, together with human chorionic gonadotropin (or its free β-subunit—free β-hCG) and ultrasound-obtained fetal nuchal translucency thickness, is used to predict the risk for fetal aneuploidies including Down syndrome [4].

To date, distributions of PlGF and PAPP-A levels in pregnant women are primarily based on Western European populations, predominantly of Caucasian ethnicity. Differences in PAPP-A and PlGF levels by ethnicity have been observed, with increased levels in Afro-Caribbean [5–14], East Asian [5,7–10,12–16], and South Asian [5,7–10,12–14,16] women, though some studies report no difference for Asian women [6,11]. Between studies ethnicity has been diversely defined, limiting study comparability. For Afro-Caribbean women it has been suggested that the difference is not constant, but increasing with gestational age. The mechanisms underlying these differences are poorly understood [6]. Ultimately, not correcting for higher levels in non-Caucasian ethnicities, especially given the known heterogeneity of people from African decent [17,18], could result in a structural underestimation of true risk for fetal aneuploidies or placental disorders [5,6].

Placenta-associated disorders in pregnancy are among the major causes of global maternal and neonatal morbidity and mortality, especially in low resource settings [19,20]. Risk prediction in early pregnancy in these settings will allow for efficient resource allocation and targeted prevention strategies including aspirin and calcium supplementation [21,22]. In addition, neonatal morbidity and mortality such as IUGR, SGA and stillbirth can be addressed concomitantly [21]. Antenatal screening programs in most resource limited settings currently focus on a specific number of diseases [23], and the possibility to expand these to predict preventable complications of pregnancy could have a substantial and cost-effective health impact—but will require a further understanding of predictive properties in this population and ethnic differences which should be taken into account.

To the best of our knowledge no study assessed the levels of PlGF and PAPP-A in pregnancy in sub-Saharan African women and compared their concentrations against existing Caucasian or Afro-Caribbean reference values. Therefore, the objective of this study is to assess the performance of existing reference values of PAPP-A and PlGF in a sub-Saharan African population in Ghana.

## Methods

### Study cohort

This study was nested in a cohort study of 1010 pregnant adult women and took place in two hospitals in Accra, Ghana (Maamobi General Hospital and Ridge Hospital Outpatient Clinic) between July 2012 and March 2014. Inclusion criteria for the present study were a singleton pregnancy with a gestational age (GA) between 56 and 97 days based on ultrasound at enrolment, maternal age more than 18 years and no known pre-existent hypertension and of whom serum was available for analysis (n = 403). Women in the cohort who had a miscarriage (n = 39), any hypertensive disorder in pregnancy (n = 91), twin pregnancy (n = 6) or fetal aneuploidy based phenotype at delivery, were excluded. Approval to conduct the study was obtained from the Ghana Health Services Ethical Review Committee (GHS-ERC 07/09/11). Written informed consent was obtained from participants prior to study inclusion.

### Study procedures

Participant demographic information including obstetric history was obtained at study inclusion by trained interviewers through structured questionnaires. Serum was obtained at day of study inclusion through venipuncture by a qualified laboratory staff member. After coagulation of the blood, it was centrifuge-spinned within two hours according to hospital protocols, and stored at -20 degrees in a temperature-controlled freezer in the central laboratory of Maamobi hospital. Serum samples obtained at Ridge Regional Hospital were stored in the fridge (4 degrees) and transported daily in a cold box with icepacks to the central laboratory in Maamobi for storage. Serum was shipped on dried-ice to the Netherlands, and stored at -80°C at the Dutch Institute for Public Health and the Environment (RIVM) laboratory until analysis in February 2014.

Obstetric and perinatal outcome information was extracted from the record books at the labor ward and postnatal clinic between 4–8 weeks postpartum. Data were double entered by two trained research assistants, and accuracy validated by merging of the two datasets and source data consultation when mistyping were flagged.

### PAPP-A and PlGF concentration measurement

Analyses of PAPP-A and PlGF were performed in a single run with a 1235 AutoDELFIA automatic immunoassay system (PerkinElmer, Turku, Finland), using automated dissociation-enhanced lanthanide fluorescent immunoassays for serum (PerkinElmer). Summarizing, proteins were eluted in wells pre-coated with anti-PAPP-A and anti-PlGF antibodies in 150 μL of buffer for 3 hours. After incubation, diluted serum was aspirated and wells washed six times. PAPP-A and PlGF concentrations were quantified using Europium (Eu) labeled tracer antibodies. After dissociation of tracer antibodies and label and chelation of the Eu ions, the amount of Eu label was measured by excitation with a 340 nm laser and detection of emitted light at 610 nm, respectively. The limit of quantification for PAPP-A is 0.04 U/L and for PlGF 7 pg/ml.

A random selection of five samples with a PAPP-A concentration of >8000 U/L were re-analyzed to exclude measurement error. Outlier assessment for implausible biomarker concentrations (>99th percentile for PAPP-A MoM >20 and PIGF MoM of >8.25) resulted in exclusion of five samples and serum of 398 participants were included in the analysis.

### Statistical analysis

Demographic and socio-economic and obstetric characteristics of the population were presented as mean and standard deviation (SD) for continuous variables and frequencies and percentages for categorical variables.

Reference equations to calculate gestational age-normalized and maternal weight-corrected PAPP-A multiple of the median (MoM) were obtained from the Dutch national prenatal screening program for Down syndrome, which includes PAPP-A measurements between 57 and 97 days of gestation for of a large population (>>10000) of Dutch women. These were;

PAPP-A:

$$y=12605.9606\;-\;552.53697x\;+\;7.42649x^{2}\;-\;0.0278x^{3},with\;x=gestational\;age\;at\;blood\;sampling\;in\;days$$

PAPP-A MoM weight correction:

y= exp(1.23234075 − 0.0181912x), with x=weight in kilograms

The reference median equation for PlGF was based on a polynominal fit of the reference values for gestational weeks 9 to 13 provided in the kit insert (PerkinElmer). The regression equation for gestation in days was:

$$y=75.08\;-\;1.7769x\;+\;0.01589x^{2},\;with\;x\;=\;gestational\;age\;at\;blood\;sampling\;in\;days$$

As in most populations PlGF concentration is not correlated with maternal weight. Thus, PlGF was not corrected for weight [24].

PAPP-A and PlGF concentrations and MoM values were presented as medians and interquartile ranges (IQR) by gestational week. For PAPP-A, weight-corrected MoM values were calculated also presented.

PAPP-A and PlGF concentrations were scatter plotted against gestational age, and a regression line was calculated using a polynominal fit, after comparison of the best fit between linear and polynominal regression using the R2 statistic. Similarly, individual PAPP-A and PlGF MoM values were plotted against weight. Correlation between PAPP-A and PlGF MoM values was calculated using the R squared statistics. Regression equations were calculated using the STATA package ‘aaplot’ [25].

Stratified analyses of PAPP-A and PlGF MoM medians and IQR were conducted for the four major ethnic groups in our population, as self-reported by participants: Akan, Hausa, Ewe and Ga-Dangme. These groups were selected from all ethnicities (>15) based on the availability of at least 30 samples. Given the sample size, difference in medians was qualitatively evaluated.

To assess possibly serum handling effects on the serum biomarker values, clustering of high MoM values by facility or date of sampling was assessed.

Statistical analysis was performed using STATA, version 11.

## Results

Serum PAPP-A and PlGF values were available for 398 women who met the inclusion criteria. Table 1 shows characteristics of included women. The mean age of participants was 28.2 years (±4.9). Mean gestational age at inclusion was 79.4 days (±11.1). The majority of women was multiparous (79.4%). The mean maternal weight was 65.3 kilograms (±12.6), mean height 161.3 (±6.0) cm. Forty-six percent of participants were overweight or obese (a body mass index (BMI) of 25 or higher). The largest ethnic groups were Akan (37.9%), followed by Hausa (17.3%), Ewe (20.6%) and Ga/Ga-Dangme (8.8%).

**Table 1:** Baseline characteristics of participating pregnant women from Accra, Ghana (n = 398).

Variable	Mean (sd), range or N = (%)
Age (years)	28.2 (4.9), 18–43
Gestational age at booking (days)	79 (11.1), 56–97
Weight (kg)	65.3 (12.6), 41.6–118.0
Height (cm)	161.3 (6.0), 142–178
BMI (kg/m2)	25.1 (4.7), 16.2–41.8
Parity	
Nulliparous	78 (19.6)
2–3 pregnancies	223 (56.0)
>4 pregnancies	97 (24.4)
BMI	
<18.5	29 (7.3)
18.5–24.99	185 (46.7)
25–29.9	124 (31.2)
>30	59 (14.8)
Ethnicity	
Akan	111 (37.9)
Hausa	69 (17.3)
Ewe	82 (20.6)
Ga, Ga-Dangme	35 (8.8)
Mole, Dagbon, Gonja and others	61 (15.3)

**Fig 1:**
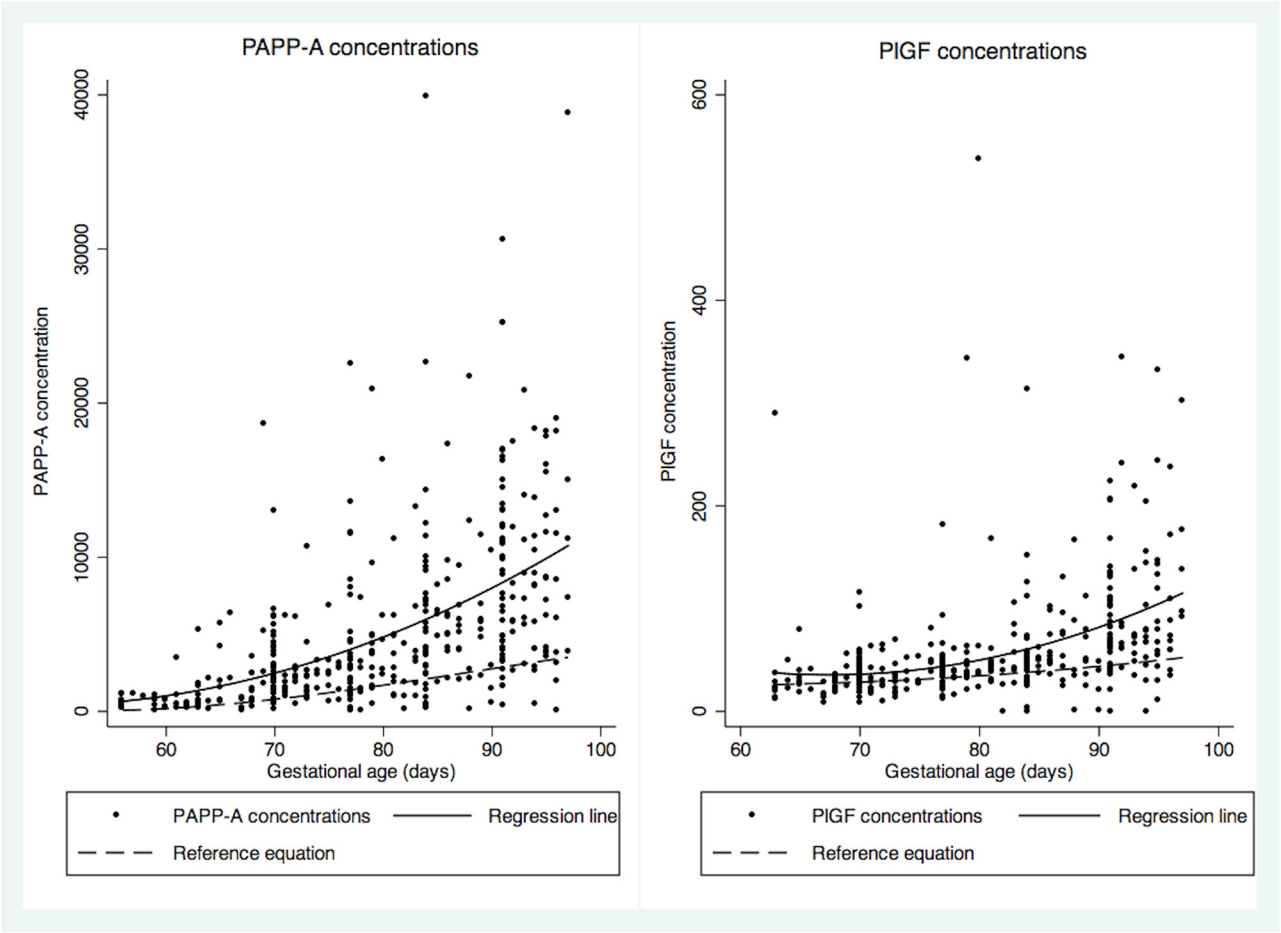
PAPP-A and PlGF concentrations scatterplot, with reference population equation and fitted regression line. Reference population equation PAPP-A: *y* = 12605.9606–552.53697*x* + 7.42649*x*^2^–0.0278*x*^3^; fitted regression line PAPP-A: *y* = 10123–408.82*x* + 4.2817*x*^2^; Reference population equation PlGF: *y* = 75.08–1.7769*x* + 0.01589*x*^2^; fitted regression line PlGF: *y* = 448.25–12.247*x =* 0.908*x*^2^.

Table 2 presents median PAPP-A and PlGF concentrations and MoM with IQR by gestational week. PAPP-A median concentration at week eight was 565 U/L (IQR 310–980 U/L) and this increased to 8240 U/L (4080–13000 U/L) at week 13. For PlGF, median concentration at eight weeks was 22.15 pg/ml (16.1–31.2 pg/ml), and this increased to 77.7 pg/ml (59.2–132 pg/ml) at week 13. Both PAPP-A and PlGF concentrations were higher in the Ghanaian population compared to the reference population (Fig 1).

**Table 2:** Median and interquartile range of concentration and MoM of PAPP-A and PlGF by gestational week (n = 398).

Weeks	N = (%)	Median concentration (IQR) PAPP-A U/L	Median concentration (IQR) PlGF pg/L	MoM PAPP-A, median (IQR), adjusted for gestational age	MoM PAPP-A, median (IQR), adjusted for gestational age and weight	MoM PlGF-A, median (IQR), adjusted for gestational age
8	31 (7.8)	565 (310–980)	22.15 (16.1–31.2)*	4.62 (1.70–8.3)	4.41 (1.57–7.90)	
9	40 (10.1)	972 (608–2285)	23.6 (19.45–31.4)	2.13 (1.32–4.75)	2.52 (1.15–4.83)	0.90 (0.74–1.20)
10	80 (20.1)	2210 (1410–3060)	32.6 (25.3–45.0)	2.20 (1.45–3.63)	2.10 (1.34–3.35)	1.14 (0.89–1.58)
11	73 (18.3)	3190 (1610–4920)	38.7 (32.7–48.6)	1.94 (0.98–3.18)	1.97 (1.06–3.20)	1.18 (0.98–1.48)
12	75 (18.8)	5120 (2830–7570)	48.8 (35.8–61.9)	2.16 (1.17–3.6)	2.25 (1.12–3.39)	1.26 (0.94–1.63)
13	99 (24.9)	8240 (4080–13000)	77.7 (59.2–132)	2.61(1.36–4.27)	2.46 (1.38–3.85)	1.73 (1.32–2.94)
Total				2.30 (1.33–4.14)	2.34 (1.24–3.97)	1.25 (0.95–1.80)

IQR = interquartile range.

* n = 34, as statistical outliers could not be excluded as PlGF MoM values were available from week 9 onwards.

Compared to the median MoM values of the reference population (1 by default), the PAPP-A median MoM was 2.30 (1.33–4.14), and 2.34 (1.24–3.97) after weight correction. The median PlGF MoM was 1.25 (0.95–1.80) (Table 3).

**Table 3:** MoM values PAPP-A and PlGF for the four main ethnic groups (n = 398 for PAPP-A, n = 365 for PlGF).

Ethnicity	N = (%)	MoM PAPP-A, median (IQR), adjusted for gestational age	MoM PAPP-A, median (IQR), adjusted for weight and gestational age	Median MoM PlGF (IQR), Adjusted for gestational age
Akan	151 (37.9)	2.63 (1.67–4.59)	2.50 (1.49–4.42)	1.26 (0.97–1.73)
Hausa	69 (17.3)	1.80 (1.07–3.18)	2.03 (1.04–2.98)	1.15 (0.81–1.65)
Ewe	82 (20.6)	2.50 (1.38–5.00)	2.46 (1.38–4.45)	1.27 (0.94–2.00)
Ga, Ga-Dangme	35 (8.79)	2.00 (0.82–4.53)	2.07 (0.88–4.21)	1.37 (0.89–1.73)

**Fig 2:**
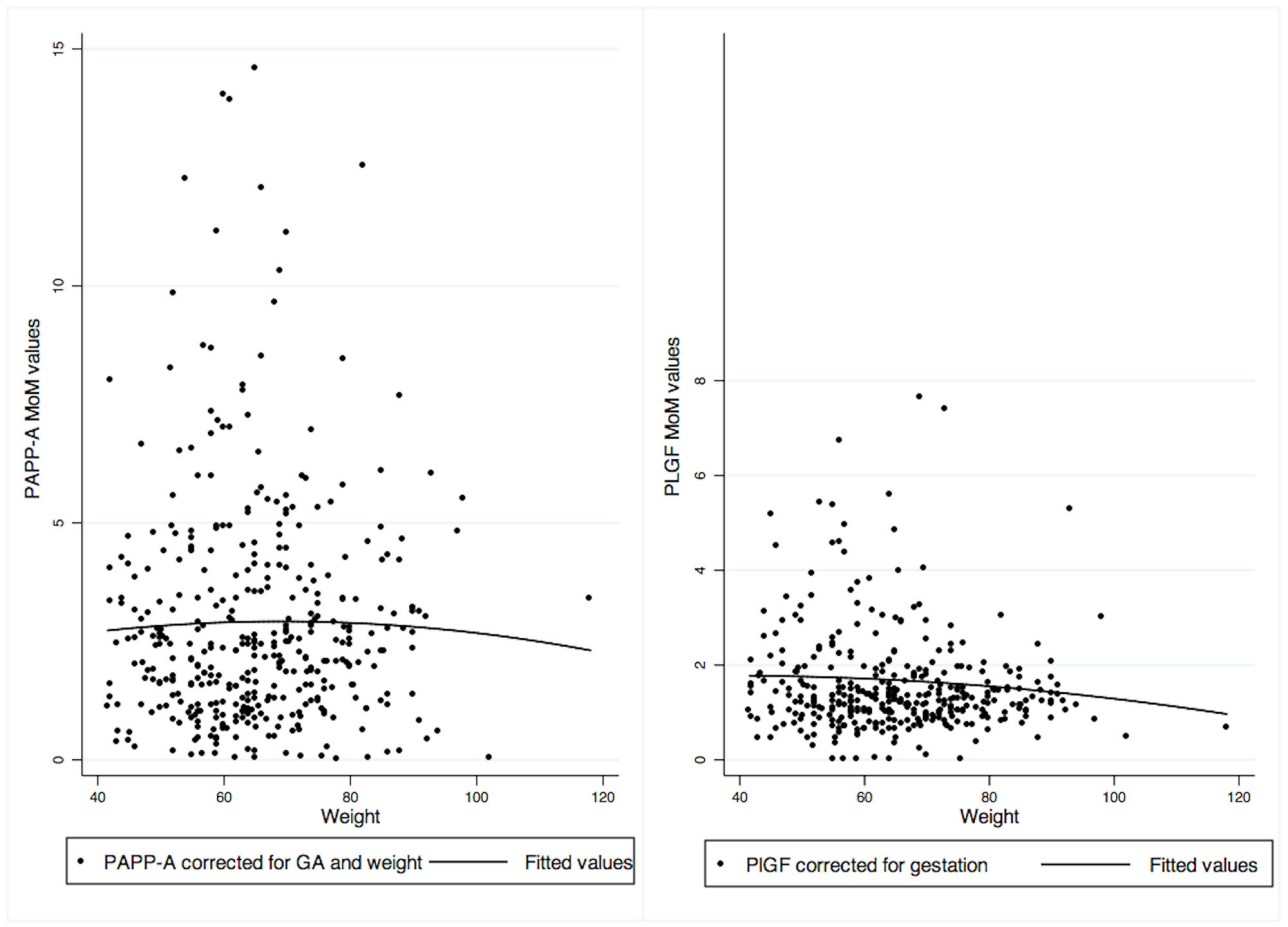
PAPP-A and PlGF MoM values with fitted regression line. PAPP-A regression equation: *y* = 1.7085 + 0.03512*x* − 0.00025*x*^2^. PlGF: *y* = 1.6012 + 0.00938*x* − 0.0013*x*^2^.

To illustrate the effect of maternal weight on PAPP-A and PlGF distribution in more detail, in Fig 2A PAPP-A MoM and weight-corrected MoM values are presented and Fig 2A PlGF MoM against weight values are presented. Weight correction resulted in slightly lower MoM values for PAPP-A (Fig 2A). PlGF MoM values remained stable across weights (Fig 2B). Correlation between PAPP-A and PlGF MoM values was low with 0.21. Clustering by facility or date of sampling was not observed for high MoM values of PlGF or PAPP-A.

For median MoM values for PAPP-A, Hausa women were at the lower end of the spectrum (GA-corrected median MoM 1.80, IQR 1.07–3.18; GA and weight corrected 2.03, IQR 1.04 to 2.98) and Akan women at the higher end (GA-corrected median MoM 2.63, IQR 1.67 to 4.59; GA and weight-corrected median 2.50, IQR 1.49 to 4.42). For PlGF values Hausa women had the lowest MoM values (median MoM 1.15, IQR 0.81 to 1.65) and Ga women were at the higher end of the spectrum (median 1.37, IQR 0.89 to 1.73).

## Discussion

In this study we observed over twice as high median MoM values of PAPP-A and a quarter higher PlGF MoM values in an urban sub-Saharan African population compared to predominantly Caucasian European population.

The PAPP-A median MoM values of our population are higher than previously observed for Afro-Caribbean women (1.46 to 1.70) [5–13]. The PlGF median MoM in our population is similar to that previously reported for Afro-Caribbean women by Padyan et al (1.30), [9] though lower than reported by Tsiakkas et al (±1.55) [14]. To exclude the possibility that our observations were a measurement error, we randomly re-analyzed samples with high concentrations, which yielded the same results. The possibility of a delay until freezing due to transportation of samples from Ridge hospital to the main laboratory at Maamobi hospital or handling at Maamobi was assessed, but clustering by facility or date of sampling was not observed for high values. Moreover, given required exposure time to (high) temperatures to induce a significant change in PAPP-A and PlGF concentrations, it is unlikely that this could explain the results [26–28]. Although the preferential rounding for gestational age determination (Fig 1) may affect the MoM when GA is over- or underestimated, this likely occurred randomly and as such would not have affected the median MoM. Therefore, the higher median MoM values in our population compared to previously reported MoM values for Afro-Caribbean women may be a reflection of the heterogeneity of the population from African decent and the differences between Afro-Caribbean population and Black Africans, as discussed by Agyemang et al elsewhere [17]. This may also explain the observed differences in PAPP-A MoM of various ethnic subgroups in our population. The difference in observed MoM values between Afro-Caribbean and Black Africans suggests the need for a specific correction factor for this population for each of the biomarkers, to avoid underestimation in the risk calculation for placental disorders or aneuploidy risk in this population. It also suggests the need to expand the MoM assessments for other sub-Saharan African populations, in order to determine what the variation is within these populations.

Placental differences in weight, volume, surface area and histology have been observed for different ethnicities [29–33], though evidence is inconclusive to determine whether this is a result of biological or environmental factors, and if this underlies the difference in PAPP-A or PlGF concentrations observed. Therefore, future research should include a better understanding of the mechanisms underlying these differences. Possible hypotheses could include higher placental production of these biomarkers, or isoform differences in antibody affinity of antigen presenting sites resulting in higher measurements without difference in absolute concentrations.

### Strengths and limitations

Strengths of this study include the design of this prospective cohort with a representative study population of urban sub-Saharan African women in a lower middle-income country, as public hospitals were included where most women receive maternal care services. The efforts in the design and follow-up are reflected in the high follow-up rate of 83%. To the best of our knowledge, this study is the first to assess MoM reference values in early pregnancy of a sub-Saharan population of PAPP-A and PlGF, as the limited number of studies conducted in sub-Sarahan Africa considered absolute serum concentrations [34–38].

Limitations of this study include the relatively low number of samples (n = 403) compared to other ethnicity studies (in high-income country setting) and our ability to control for some known confounders. This, combined with the inherent bias in gestational age determination in early pregnancy reflected in the preferential rounding, may also explain the high median MoM at week 8, and the absence of a clear upward trend of median PAPP-A MoMs from week 9 to week 13, which is usually observed for PAPP-A. Information about smoking was not available, but unlikely to have affected the results due to the low prevalence among Ghanaian women (0.3%) [39]. Similarly, although aneuploidies were excluded from the analysis, this was based on clinical assessment and not karyotyping. However, given the low incidence of trisomies (<1/1000 live births) [40,41], this will likely not have effected the MoM estimates. Information about method of conception including assisted reproductive technique used was also not available, but expected to be sporadic at best in the participating low resource public hospitals [42]. As in especially middle-income countries assisted reproductive techniques are increasingly available, the effects of these on pregnancy-related biomarkers will be important to consider in the future [42].

### Clinical implications

Prenatal screening programs for fetal aneuploidies or placental disorders using biomarkers PAPP-A and PlGF are available in most high resources settings, and increasingly available in low- and middle-income countries. Given the global migration patterns and (increasingly) diverse background of pregnant women [43], ethnicity should be considered in planning and execution of prenatal screening programs.

The global burden of maternal and neonatal morbidity and mortality associated with placenta-related disorders such as pre-eclampsia, IUGR and SGA require innovative strategies to optimize antenatal care in resource-constrained settings [19,20,44]. Stratification in antenatal care delivery and targeted interventions for high-risk pregnancies, are a promising approach, but will require the evaluation of predictive properties of in a specific population before implementation.

## Conclusion

PAPP-A and PlGF MoM values appear to be substantially higher in a sub-Saharan African population compared to existing reference values. The difference in observed MoM values between Afro-Caribbean and Black Africans suggests the need for a specific correction factor for this population, to avoid underestimation in the risk calculation for placental disorders and fetal aneuploidy.
